# Development of an in-vitro high-throughput screening system to identify modulators of genitalia development

**DOI:** 10.1093/pnasnexus/pgac300

**Published:** 2022-12-28

**Authors:** Yan Yin, Meade Haller, Tian Li, Liang Ma

**Affiliations:** Division of Dermatology, Department of Medicine, Washington University School of Medicine, 660 S. Euclid Ave., St. Louis, MO 63110, USA; Division of Dermatology, Department of Medicine, Washington University School of Medicine, 660 S. Euclid Ave., St. Louis, MO 63110, USA; Division of Dermatology, Department of Medicine, Washington University School of Medicine, 660 S. Euclid Ave., St. Louis, MO 63110, USA; Division of Dermatology, Department of Medicine, Washington University School of Medicine, 660 S. Euclid Ave., St. Louis, MO 63110, USA; Department of Developmental Biology, Washington University School of Medicine, 660 S. Euclid Ave., St. Louis, MO 63110, USA; Department of Obstetrics and Gynecology, Washington University School of Medicine, 660 S. Euclid Ave., St. Louis, MO 63110, USA

**Keywords:** external genitalia, genital tubercle mesenchyme, proliferation, high-throughput screening, VEGFR

## Abstract

Sexually dimorphic outgrowth and differentiation of the embryonic genital tubercles (GTs) give rise to the penis in males and the clitoris in females. Defects in androgen production or in response to androgen signaling can lead to various congenital penile anomalies in both mice and humans. Due to lack of a high-throughput screening system, identification of crucial regulators of GT sexual differentiation has been slow. To overcome this research barrier, we isolated embryonic GT mesenchymal (GTme) cells to model genitalia growth and differentiation in vitro. Using either a mechanical or fluorescence-activated cell sorting–assisted purification method, GTme cells were isolated and assayed for their proliferation using a microscopy and image analysis system, on a single cell level over time. Male and female GTme cells inherently exhibit different cellular dynamics, consistent with their in-vivo behaviors. This system allows for the rapid quantitative analyses of numerous drug treatments, and enables the discovery of potential genetic modulators of GT morphogenesis on a large scale. Using this system, we completed a 438-compound library screen and identified 82 kinase inhibitor hits. In mice, in-utero exposure to one such candidate kinase inhibitor, Cediranib, resulted in embryos with severe genitalia defects, especially in males. Gene silencing by RNAi was optimized in this system, laying the foundation for future larger-scale genetic screenings. These findings demonstrate the power of this novel high-throughput system to rapidly and successfully identify modulators of genitalia growth and differentiation, expanding the toolbox for the study of functional genomics and environmental factors.

Significance Statement Congenital anomalies of the genitalia are under-reported and heavily stigmatized. They have a combined incidence of 1 in 120, can be caused by genetic or prenatal environmental factors, and have limited treatment options. Previous studies identifying causes for genitalia developmental anomalies focus on single genes, single compounds, or at most, single pathways. The novel high-throughput screening system described herein can rapidly screen hundreds of potential modulators of mesenchymal growth and differentiation in the genitalia, representing a powerful tool to better identify the etiologies of genital anomalies, and ultimately diversify treatment options. Additionally, this system can easily be adapted to study developmental and disease biology of numerous organ systems.

## Introduction

Mammalian external genitalia are structures of the reproductive system that exhibit remarkable sexual dimorphism. They are composed of the labia minora and majora and the clitoris in females, and penis and scrotum in males. The genital tubercle (GT), the embryonic anlage of the external genitalia, undergoes a sex-independent early outgrowth stage and an androgen-dependent sexual differentiation stage [for review, see ref. (1)]. In mice, the GT starts to emerge as early as embryonic day 10.5 (E10.5) as a thickened epithelial structure covering the posterior-ventral side of the cloaca, consisting of an epidermal epithelium and an underlying endodermal-derived cloacal epithelium (2). The convergence of these two epithelial structures creates a signaling center termed the distal urethral epithelium (dUE), which provides morphogens to guide subsequent outgrowth and differentiation of both epithelial and peri-cloacal mesenchymal compartments (2–8). By E14.5, GTs are morphologically indistinguishable between sexes and are composed of a proximal region surrounded by preputial swellings and a distal region of exposed glans. At E14.5, the initial sex-independent developmental stage has completed (9). From E15.5, escalating levels of circulating androgens in male embryos promote GT masculinization via continuous growth and extensive remodeling of the GT mesenchyme and epithelia, resulting in a penis at birth with an entirely internalized urethra that opens distally at the tip of the glans (10). By contrast, the female GT undergoes less extensive development, and the urethra terminates proximally at the base of the clitoris. Inactivating androgen receptor (AR) signaling with either genetic ablation of the receptor gene or pharmacological inhibitors results in clitoris formation in male mice (11, 12). Estrogen receptor (ER) signaling appears to antagonize and balance AR signaling, as exposure to exogenous estrogen prevents penile outgrowth in males, while *Esr1* knockout females develop longer urethra and larger external genitalia (13).

Congenital anomalies of the external genitalia represent one of the most prevalent congenital phenotypes in humans, affecting approximately 1 in every 120 male newborns (14). Hypospadias, a structural anomaly with ectopic ventral urethral opening, is estimated to affect 1 in every 200 infant boys born in the United States (15). Penile hypoplasia or micropenis, a condition where the penis is at least 2.5 SD shorter than the age-matched average, is found in 0.015% of male infants in the United States (14). Congenital penile anomalies such as hypospadias and micropenis are often viewed as loss or reduction of male-typic morphology. The etiology for these conditions usually remains unknown, but many believe that it reflects developmental disruptions that occurred during sex differentiation of the GT, involving genetic, hormonal, and/or environmental elements. Most of the current knowledge of GT development and pathologies was obtained by studying genetic animal models or in-utero exposure to environmental factors. Recent advancements in single cell RNA sequencing have helped identify numerous genes that specify cell-type identities and regulate GT differentiation (16, 17), but further characterization of their roles in GT outgrowth and development could be time consuming in the absence of a rapid high-throughput screening system.

Here, we report the development of an isolation protocol where either enriched or pure GT mesenchymal (GTme) cells are obtained from mouse embryos, and the establishment of an image-based proliferation assay that monitors both their cellular proliferation and differentiation. Using this protocol, sex-dimorphic growth was observed in GTme cells in vitro, and the system was adapted to rapidly identify genetic and chemical modulators of GT development.

## Results

### Isolation, characterization, and visualization of in-vitro proliferation of GTme cells

**Fig. 1.**
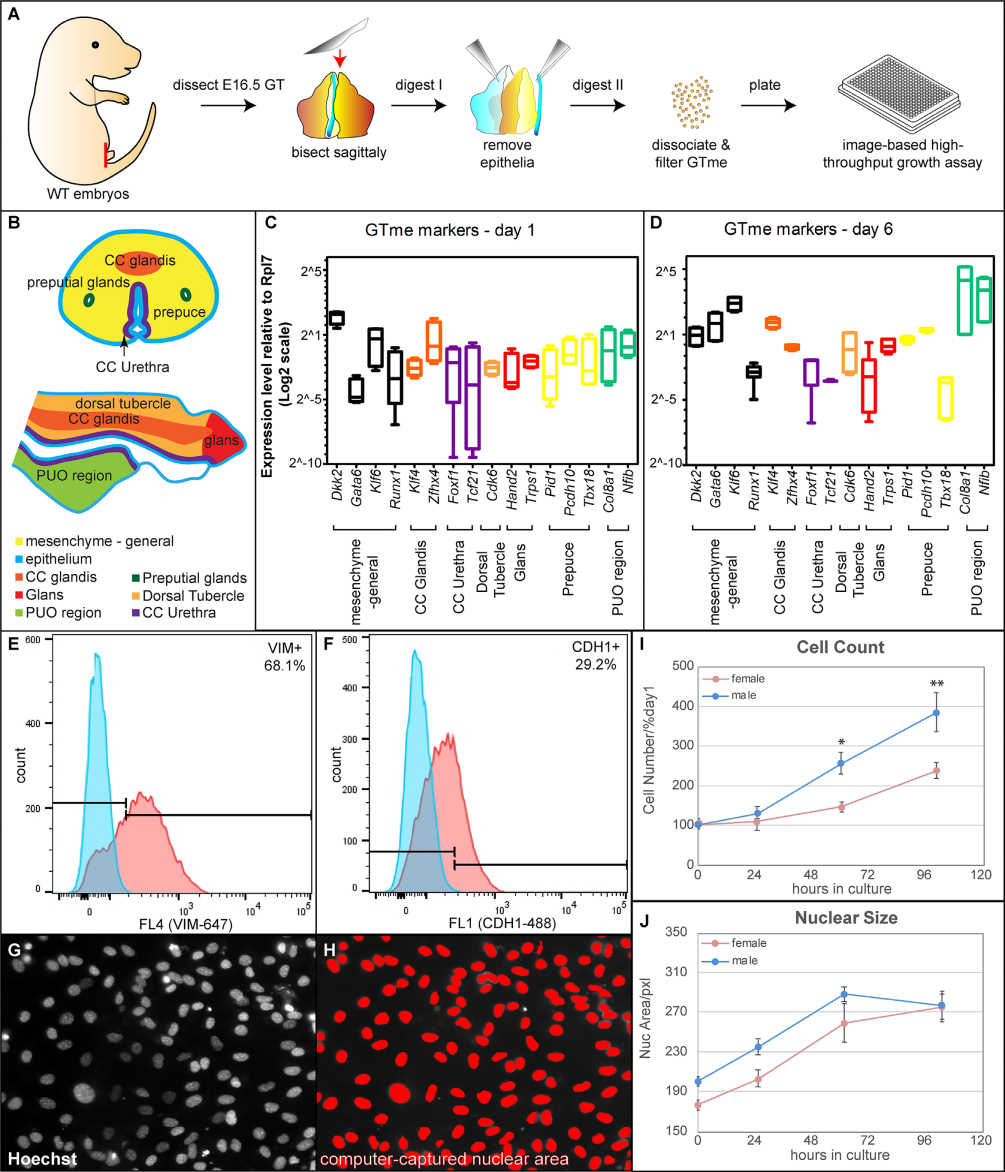
Isolation and in-vitro proliferation of WT GTme cells. (A) Schematic illustration of the GTme isolation process from WT embryos. (B) Transverse and sagittal diagrams of the subpopulations of E16.5 GT (adapted from Armfield and Cohn). (C and D) Gene expression of mesenchymal and subpopulation markers in GTme cells after 1 day (C) and 6 days (D) in culture, assayed by real-time RT-PCR. (E and F) Representative histograms of the FACS of isolated GTme cells stained with antibodies against mesenchymal marker VIM (E) and epithelial marker cadherin1 (CDH1, F), respectively; blue, isotype control; red, antibody-stained. (G and H) Fluorescence microscopic image of Hoechst-stained GTme nuclei (G) was analyzed by IN Cell Developer to computationally capture individual nucleus for accurate counting and measuring (H). (I) Growth curves and (J) average nuclear size curves of GTme cells in culture. *P < 0.05; **P < 0.01.

One of the earliest cellular and morphological differences after the onset of GT sexual differentiation is mesenchymal cell proliferation. Under androgen signaling, a much higher proliferation index was observed in male GTs than in female GTs (3, 18). We reasoned that this sex-specific difference in cell proliferation could potentially be used as a readout for a high-throughput screening assay. However, it was not known whether this sex-specific mitotic difference persists after the cells are placed in culture. To test this, wild-type (WT) embryonic day 16.5 (E16.5) mouse GTme cells were isolated by sequential enzymatic digestions and mechanical separation (Figs. 1A and S1). At E16.5, the GT has recently exited the bipotential stage of development (E14.5) and entered sexual dimorphic differentiation driven by testis-derived androgens into penile tissue in males, and in females into clitoral tissue in the absence of testosterone (19). Having been exposed for at least 2 days to endogenous androgens, the male GTme cells are expected to be preprogrammed to proliferate at higher rates than female cells. The size of E16.5 GT is relatively indistinguishable between male and female, but large enough to manipulate tissue layers and to yield substantial numbers of cells per GT in both sexes. Isolated GTme cells formed a single layer on poly-d-lysine–treated culture plates overnight and grew rapidly for 10 to 14 days in vitro. To evaluate the nature of the isolated cells, quantitative RT-PCR and fluorescence-activated cell sorting (FACS) were performed to detect GTme markers in GTme cells. Based on recent studies, utilizing single cell RNA sequencing on developing mouse GT cells, we surveyed expression of both general mesenchymal markers as well as those specific to subpopulations by either FACS or quantitative RT-PCR, both at the beginning and end of the in-vitro culture system. General mesenchymal markers, Vimentin (VIM), *Dkk2, Klf6, Gata6*, and *Runx1*, were detected in abundance in these cells [Fig. 1B to E, (16)]. Markers for every subpopulation of GTme (16), including the corpus cavernosum (cc) glandis (*Klf4, Zfhx4*), cc urethra (*Foxf1, Tcf21*), dorsal tubercle (*Cdk6*), glans (*Hand2, Trps1*), prepuce (*Pid1, Pcdh10, Tbx18*), and the PUO region (*Col8a1, Nfib*), were all detected at various levels (Fig. 1C and D). Interestingly, toward the end of the culture, even though expression of the majority of these markers remained at similar levels, expression of both PUO region markers were markedly elevated (Fig. 1D). It is noteworthy that GTme cells isolated with this method also contained a small fraction of epithelial cells (endodermal- and/or epidermal-origin), evidenced by CDH1 expression (Fig. 1F). Therefore, this enzymatic and mechanical isolation method only provides an enrichment of GTme cells, as opposed to a pure population, and this issue is addressed later in the study using a refined FACS-based isolation method. Nevertheless, since the vast majority of the isolated cells were GTme cells, and because the use of wild-type mice makes the isolation both rapid and widely accessible to other researchers, we proceeded with cells extracted by this method for the initial experiment setup, optimization, and proof-of-concept screening.

To quantify GTme cell proliferation, we adopted a high-throughput, large-scale measurement method at the single cell level based on our previous screening study using an immortalized cell line (20). Using 384-well plates with quadruplets for each condition, we were able to image fluorescently labeled nuclei (Fig. 1G, white), computationally capture and measure the size of individual nuclei, as well as count the total number of cells in each well (Fig. 1H, red). The image-based proliferation assay not only accurately reflected the number of cells, but also recorded the morphology of the nucleus, as an indicator of cell viability and function. Intriguingly, a time-course proliferation study revealed a sexual-dimorphic growth pattern of GTme cells in vitro (Fig. 1I). GTme of male origin exhibited a growth advantage over female counterparts over a period of 5 days, indicating intrinsic activation of the AR signaling pathway in the male GTme, which sustains the proliferation of these cells at a higher rate. The average nuclear size of male GTme cells was slightly larger than that of females at the beginning of culture, and the nuclear size in both sexes increased over time and eventually plateaued where they converged (Fig. 1J). We observed a negative correlation between proliferative capacity and nuclear size. As the nuclear size reached a threshold past which it no longer increased (mere passage 3 cells), this is the same timepoint at which proliferation substantially slowed (Fig. S2A and B, compared to Fig. 1I and J). Thus, we speculate that the observed increase in nuclear size is positively correlated with cell differentiation. We have previously observed this correlation in human endometrial stromal cell during decidual induction in vitro (20). This notion was also corroborated by the evidence that established cell lines (EM-TERT, an immortalized normal human endometrial cell line, and AN3CA, a human endometrial carcinoma cell line) with constant growth rate over many passages without differentiation did not exhibit any changes in their nuclear size over time (Fig. S2C and D). Taken together, we were able to isolate GTme cells from wild-type mouse embryos during sexual-dimorphic development and show sexual-dimorphic GTme cell proliferation in vitro.

### Identification of kinase inhibitors regulating GTme proliferation

**Fig. 2.**
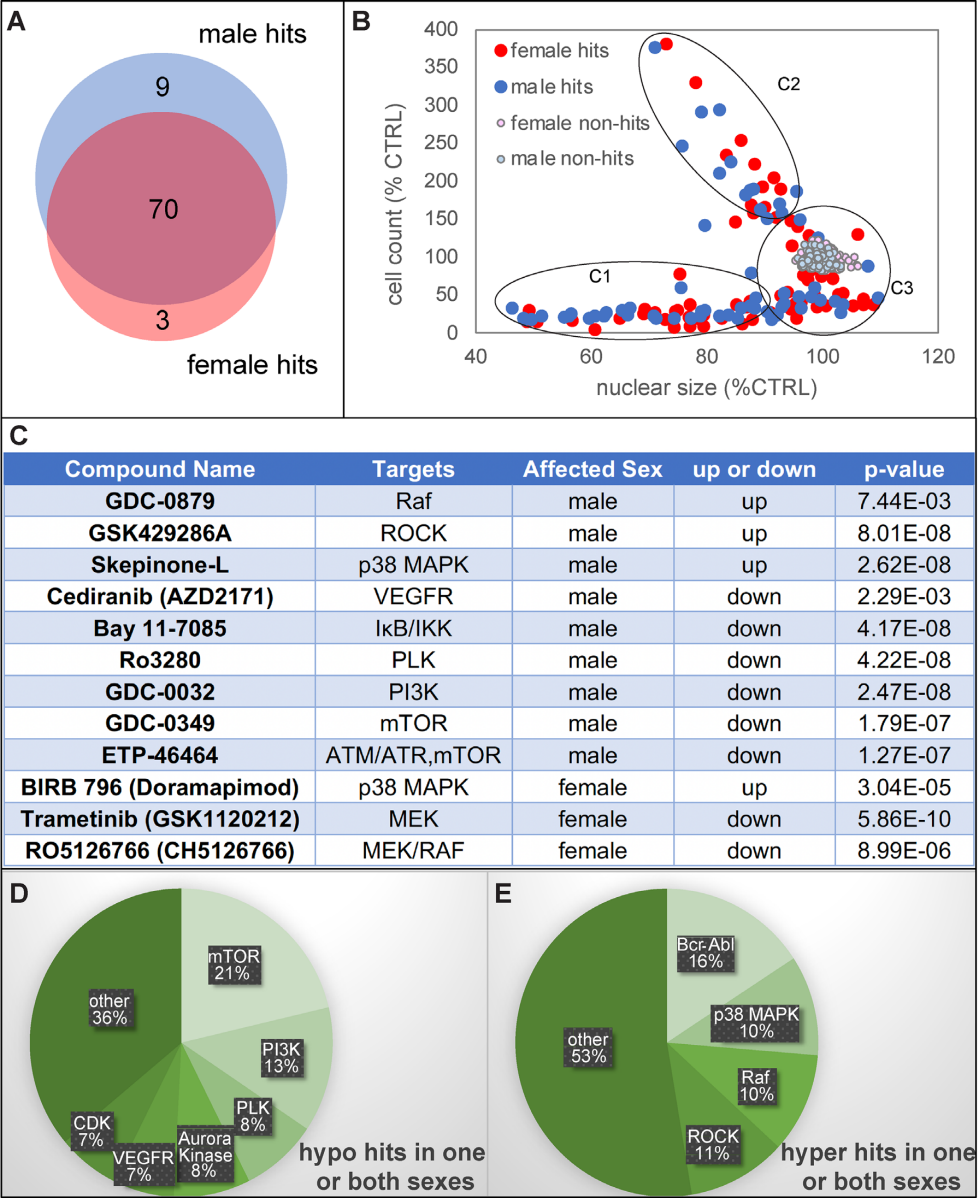
Identification of kinase inhibitors that affect GTme growth in vitro. (A) Venn diagram of the 82 hits. (B) Scatterplot of all CPDs on cell count vs. nuclear size categorizing all hits into three groups (C1, C2, and C3). (C) List of hits that exhibited a sex-dimorphic response in GTme proliferation. (D and E) Functional clusters of hypo and hyperhits.

The establishment of GTme cell culture allowed us to develop a high-throughput screening tool to identify genes and pathways regulating GTme proliferation. As a proof-of-concept experiment, we performed a screen to determine factors that modulate GTme cell growth with the Selleck© Chemicals kinase inhibitor library. This library contains 438 unique structurally diverse and cell permeable compounds (CPDs), targeting kinases that are involved in a plethora of cellular processes. Each library plate contains 80 unique CPDs, which were used to treat two 384-well assay plates for both sexes in technical quadruplets at a final concentration of 250 nM. Each assay plate also includes DMSO-treated negative controls to assess vehicle toxicity. At the end of 6 days in culture, each plate was imaged after counter-staining with Hoechst dye on the automated IN Cell Analyzer 2,000 imaging system. Captured images were analyzed in IN Cell Developer toolbox, which exported total cell count and average nuclear size of each well in an Excel file. Median absolute deviation (MAD) of cell count was calculated for each plate, and a CPD was considered a hit if the average cell count per well of its technical quadruplets was 3MAD or more away from the median cell count per well of that plate, in either sex. A total of 82 CPDs met these criteria, and the vast majority of them exhibited similar effects in both sexes (Fig. 2A, Table S1). Nine compounds affected male GTme specifically, while three affected female GTme specifically (Fig. 2A and C). Seven out of the 160 negative control wells showed up as hyperhits, making the false discovery rate (FDR) for hyperhits 4.375%, and the FDR for hypohits below the threshold of detection.

As this method allowed us to measure nuclear morphology in addition to cell number, we plotted cell count against nuclear size and discovered that the hits roughly fell into three categories (Fig. 2B): C1, CPDs that inhibited cell proliferation as well as causing nuclear shrinkage/fragmentation; C2, CPDs that promote cell proliferation while causing nuclear shrinkage; and C3, CPDs that affect cell proliferation in either direction while having minimal impact on nuclear morphology. C1 CPDs clearly indicated their cytotoxic nature, while CPDs in C2 and C3 needed to be studied in a case-by-case manner. Close examination of the hypohits (hits that impair proliferation) revealed that most of them are inhibitors targeting kinases that are critical for cell cycle progression, including mammalian target of rapamycin (mTOR), PI3K, polo-like kinase (PLK), Aurora kinases, and CDKs (Fig. 2D); and hyperhits (those that enhance proliferation) were composed mainly of Bcr-Abl, p38 MAPK, RAF, and ROCK inhibitors (Fig. 2E). PANTHER pathway ontology analyses on all the target genes of the kinase inhibitor hits revealed that angiogenesis, CCKR signaling, and VEGF signaling pathway are the top three statistically overrepresented pathways (Table S2). Taken together, by using the crude GTme isolation method coupled with the image-based proliferation assay, we were able to rapidly identify small molecules that affect GTme proliferation in vitro.

### Generation of a mesenchymal-specific Cre mouse model and refinement of the GTme isolation procedure

**Fig. 3.**
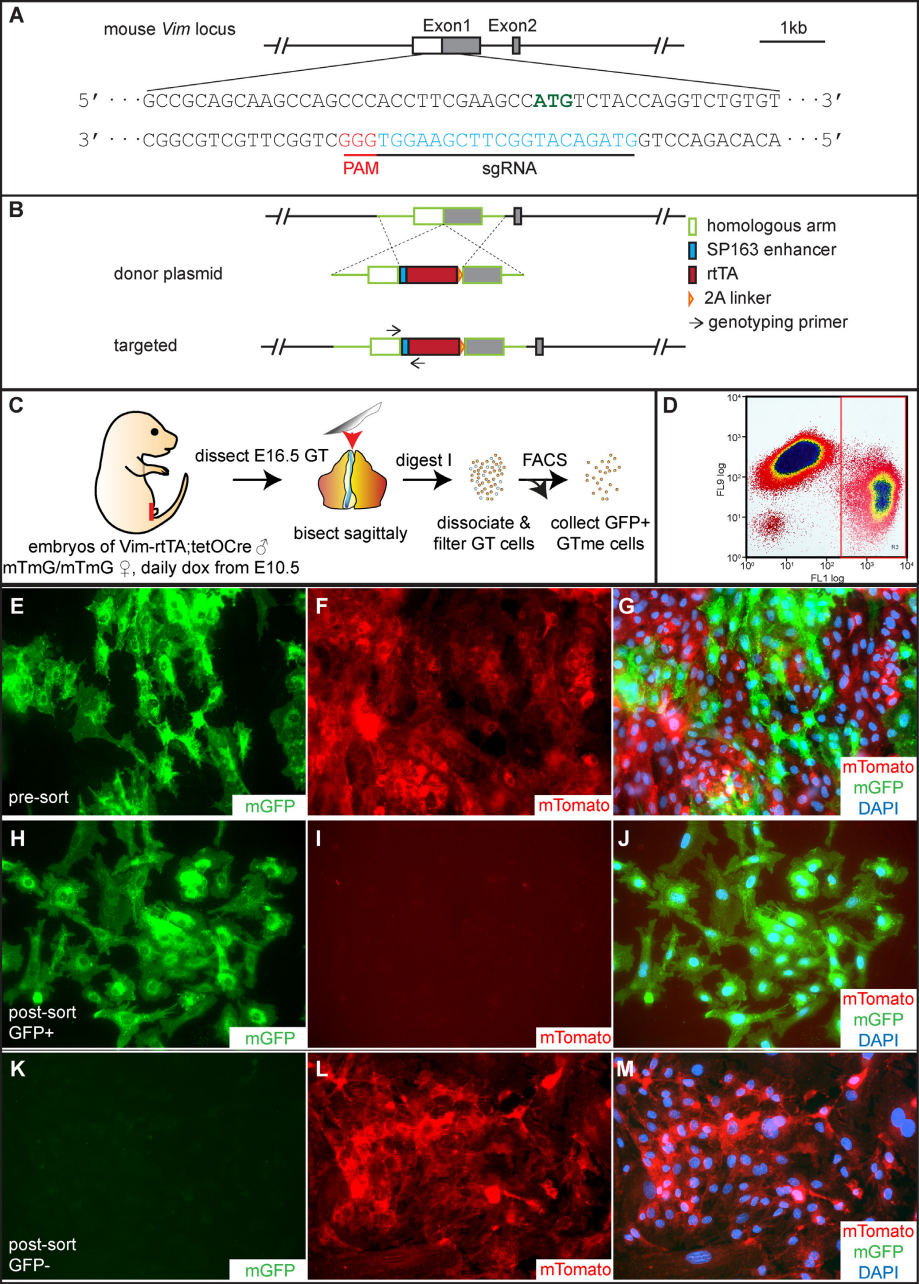
Generation of *Vim*-rtTA mouse and isolation of mTmG*^Vim^* GTme cells. (A) Location and sequence of sgRNA in relation to the mouse *Vim* genomic locus. (B) Schematics of donor plasmid design, homologous recombination event, and targeted *Vim* allele. (C) Schematic illustration of the GTme isolation process from mTmG*^Vim^* embryos. (D) Representative FACS dot plot showing separation of green GFP+ (FL1) and red Tomato+ (FL9) populations. (E to M) Fluorescence microscopy of GTme cells before (E to G) and after sorting (H to J, gated for GFP; I to M, gated for Tomato). Cells were allowed to attach to cover glass in culture before imaging.

Since isolating wild-type GTme cells unavoidably contains a small portion of epithelial cells, we sought to establish a mouse model that could improve the purity of the isolated cell population, while at the same time simplifying the isolation procedure. To do this, we first need to establish a driver that would enable gene manipulation specifically and efficiently in the embryonic GT mesenchyme. We selected the mouse vimentin (*Vim*) gene as a knock-in target, as it has been extensively shown to be expressed in a variety of mesenchymal tissues including the GT mesenchyme. For efficient knock-in, we chose the CRISPR-Cas9 system that would cut DNA in the immediate vicinity of the *Vim* start codon with high on-target cleavage activity in the presence of sgRNA (Figs. 3A, S3A, and B). A donor plasmid containing two homologous arms targeting the insertion sites flanking a SP163 enhancer, an rtTA coding sequence, and a 2A linker was constructed (Fig. 3B). The SP163 enhancer can increase translational efficiency of the inserted rtTA, while the 2A linker allows simultaneous expression of rtTA and the endogenous *Vim* gene. Successful genomic engineering events were confirmed first in cultured mouse N2A cells in the presence of sgRNA, donor plasmid, and Cas9 (Fig. S3C), before microinjection into fertilized mouse eggs. One *Vim-*rtTA founder was obtained after two rounds of injection, and the founder showed expected Mendelian ratios in F1 offspring (Fig. S4A). The *Vim*-rtTA founder was subsequently bred to tetO-Cre transgenic mice, which express Cre recombinase in a doxycycline (Dox)-rtTA-dependent manner (21). To evaluate the spatial and temporal control conferred by this inducible system, studs carrying both *Vim*-rtTA and tetO-Cre were used to mate to the well-characterized ROSA26^mTmG^ reporter females treated with Dox during gestation [(22) Fig. S4B]. In embryos carrying *Vim*-rtTA, tetO-Cre as well as one allele of ROSA26^mTmG^ (hereafter referred to as mTmG*^Vim^*), rtTA is expressed in all *Vim*-expressing cells but does not activate Cre expression in the absence of Dox. When the pregnant dam receives Dox administration, rtTA undergoes a conformational change, which enables its binding to the tetO promoter and activation of the tet-responsive element and Cre expression, which in turn excises the floxed tdTomato gene at the *ROSA26* locus and allows mGFP expression (Fig. S4C). Immunofluorescence (IF) staining of GFP in a postnatal day 2 (P2) mTmG*^Vim^* mouse GT clearly illustrated its exclusive and extensive expression in the mesenchyme (Fig. S4D, CDH1 labels endoderm-derived urethra and ectoderm-derived epidermis). Expression of GFP overlapped with that of VIM in the GT (Fig. S4E and F), and was detected in other mesenchymal tissues such as mesenchyme of the epididymis and kidney (Fig. S4G and H). The only exception is the uterus, where GFP-positive cells were also detected in the luminal epithelium (Fig. S4I, arrows). This finding supports the notion that the embryonic origin of the uterine lining is partially through mesenchymal–epithelial transition (MET) in the underlying stroma, as previously reported (23).

With this genetic tool in hand, we simplified our GTme isolation procedure to one enzymatic step followed by cell dissociation and FACS sorting based on cellular fluorescence (Fig. 3C). The mating scheme will result in one quarter of the embryos carrying all three alleles (*Vim*-rtTA, tetO-Cre, and mTmG). When GT cells were pooled together by sex, all GFP+ cells would be mesenchymal cells from mTmG*^Vim^* embryos due to *Vim*-mediated Dox-dependent Cre activation, whereas the mTmG*^Vim^* epithelial cells as well as all cells from non-triple-transgenic embryos would remain red. This expectation was confirmed by FACS, where a mixture of mTomato+ and mGFP+ cells were observed prior to sorting (Fig. 3E to G), which were clearly separated into two populations by FACS (Fig. 3D) and pure GFP+ GTme cells were obtained after sorting (Fig. 3H to M). Based on the percentages of GFP+ cells acquired from each sorting, we estimated that up to 70% of each mTmG*^Vim^* GT were GTme cells, yielding ∼100,000 to 200,000 cells.

**Fig. 4.**
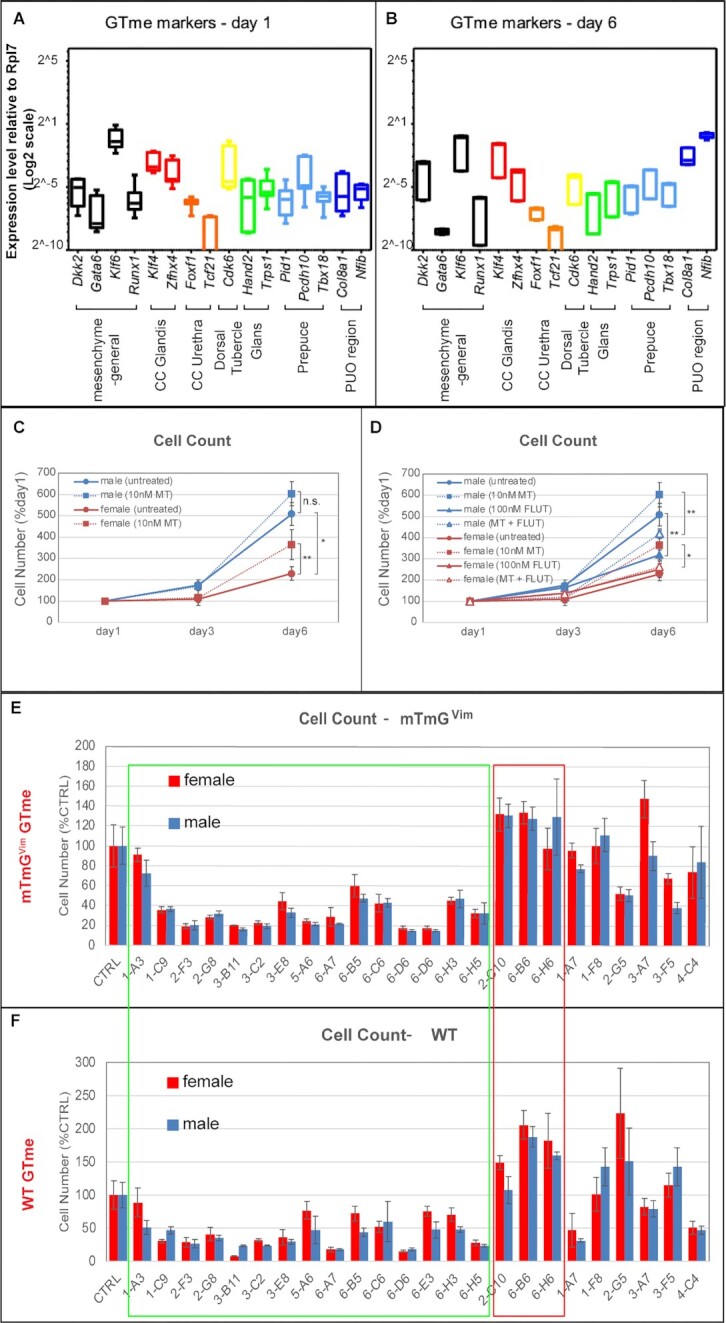
Characterization and in-vitro proliferation of mTmG*^Vim^* GTme cells. (A and B) Gene expression of mesenchymal and subpopulation markers in mTmG*^Vim^* GTme cells after 1 day (A) and 6 days (B) in culture, by real-time RT-PCR. (C and D) Growth curves of mTmG*^Vim^* GTme cells, with or without treatment of MT and/or FLUT. *P < 0.05; **P < 0.01. (D and E) Comparison of 24 kinase inhibitor hits on proliferation of mTmG*^Vim^* (D) and WT GTme cells (E). Green box outlines hypohits with similar responses in both cell lines and red box outlines hyperhits with similar results.

Similar to their WT counterparts, markers for all subpopulations of developing GT were detected in mTmG*^Vim^* GTme cells, with those of PUO regions slightly increased over the period of culture (Fig. 4A and B). Time-course growth assay was performed on mTmG*^Vim^* GTme cells and similar sex-specific growth patterns were observed where male GTme exhibited a higher proliferation index (Fig. 4C). Intringingly, supplementation of 10 nM methyltestosterone (MT) in culture media significantly enhanced proliferation of female GTme cells, whereas a similar trend was observed in male cells although not statistically significant (Fig. 4C). Treatment of an AR signaling antagonist, flutamide (FLUT), on the other hand, attenuated male GTme growth markedly without affecting female GTme proliferation (Fig. 4D). Co-treatment of FLUT and MT, significantly reduced cell growth in both sexes, when compared to MT-treatment alone (Fig. 4D). These data demonstrated that the sex-dimorphic proliferation exhibited by isolated GTme cells is androgen-driven and mimic the behaviors of these cells in vivo. We randomly picked 24 of the kinase inhibitor hits (about one-third of all hits) and repeated the screening on mTmG*^Vim^* GTme cells. Similar promoting/inhibitory effects were confirmed in mTmG*^Vim^* GTme cells for 18 out of 24 tested CPDs (Fig. 4E compared to 4F). Green box outlines the 15 hypohits that repeated in mTmG*^Vim^* GTme cells, and red box outlines the 3 repeated hyperhits. The discrepancy in the screening results may be caused by the presence of epithelial cells in the WT GTme culture and differential cellular responses of these cells to the CPDs. Taken together, the refined system allowed us to simplify the GTme isolation process, purify GTme cells with little if any contamination of other cell types, and pave the way for large-scale genomic and chemical screening for modulators of GTme proliferation in the future.

### Validation of a candidate hit in vivo

**Fig. 5.**
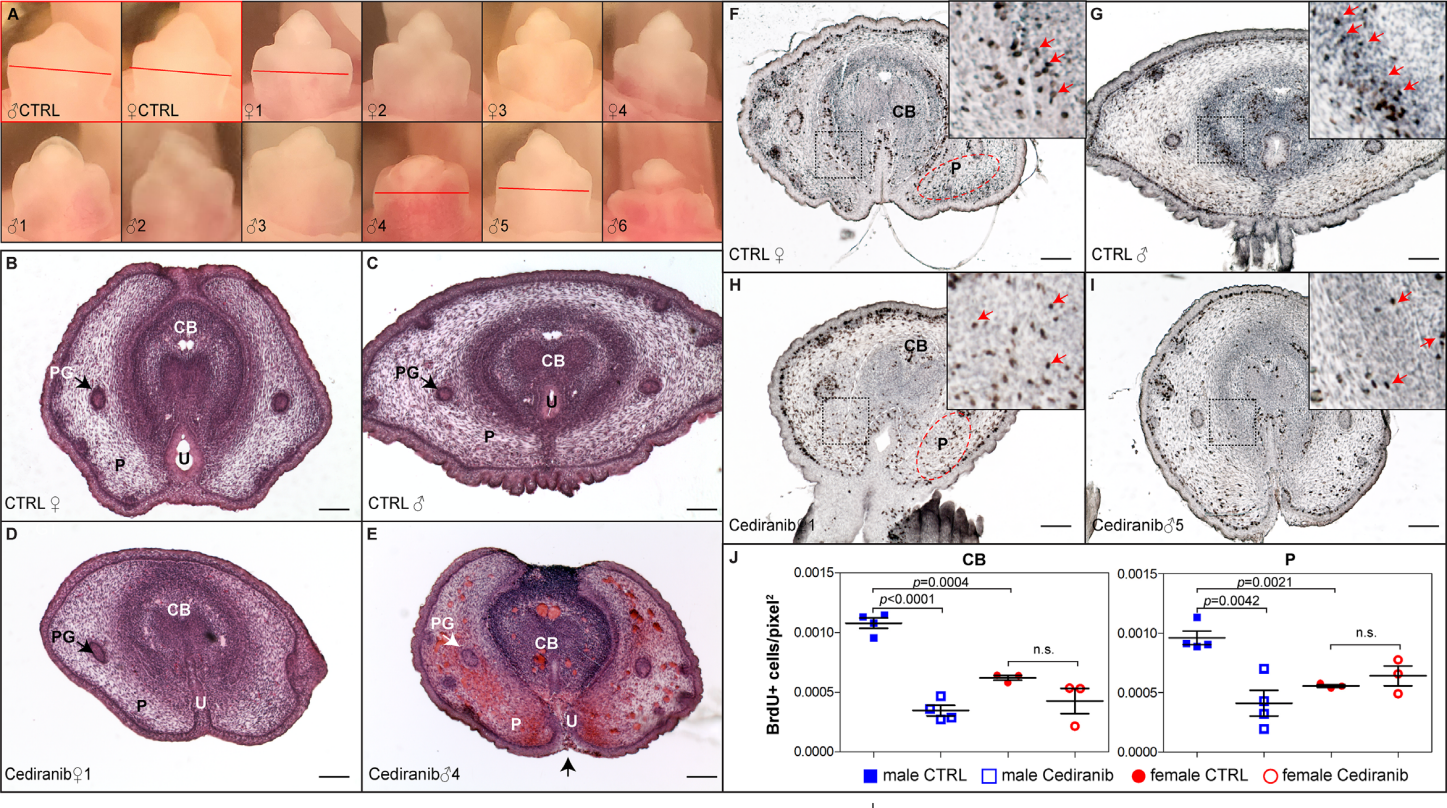
Adverse effect of Cediranib on male GT development in vivo. (A) Gross morphology of control E17.5 GTs (red outline), and that of a litter of 10 pups treated daily with Cediranib from E14.5 to E16.5. Red lines indicate planes of sections shown in B to I. (B to E) H&E staining of transverse sections of control- and Cediranib-treated E17.5 GT. PG, preputial glands; P, prepuce; U, urethra. (F to I) BrdU staining of transverse sections of control- and Cediranib-treated GT. Insets showed magnified view of boxed areas. Red arrows point to BrdU+ cells. (J) Quantification of BrdU+ cells per unit area in corpus body (white-dotted regions in F and H) and prepuce (red-dotted regions in F an H). CB, CTRL male 0.001079 ± 4.346e-005, *N* = 4 vs. Cediranib male 0.0003480 ± 4.444e-005, *N* = 4, *P *< 0.0001; CTRL female 0.0006211 ± 2.044e-005 *N* = 3, vs. Cediranib female 0.0004279 ± 0.0001056, *N* = 3, *P *= 0.1467; P, CTRL male 0.0009609 ± 5.790e-005 vs. Cediranib male 0.0004110 ± 0.0001081, *N* = 4, *P *= 0.0042; CTRL female 0.0009609 ± 5.790e-005, *N* = 4 vs. Cediranib female 0.0005567 ± 1.158e-005, *N* = 3, *P *= 0.14.

The goal of the GTme in-vitro screening system is to discover factors that could also affect GT growth and differentiation in vivo. As a proof-of-concept experiment, we chose one candidate, Cediranib, to test its effect on GT development in vivo. Cediranib is a potent vascular endothelial growth factor receptor (VEGFR) inhibitor that was developed as a potential oral antineoplastic to treat a variety of cancer types due to its antiangiogenic nature [for review, see ref. (24)]. In the screening using both WT GTme and mTmG*^Vim^* GTme cells, Cediranib exhibited an inhibitory effect on GTme cell proliferation more prominently in cells of male origin. To test its effect in vivo, pregnant WT dams received daily oral gavage of Cediranib [5 mg/kg b.w., (25)] from E14.5 to E16.5, and embryos were harvested on E17.5 to assess GT development. Cediranib treatment of six dams led to preterm labor in three animals, but did not affect parturition in the other three, possibly due to the “vascular normalization effect” elicited by Cediranib and variant individual tolerance to the drug (26). Comparable number of pups and fetal weight were observed in the litters of these three dams (Fig. S5A to F). There was also no difference in free testosterone levels in either plasma or testicular lysates when comparing vehicle- and Cediranib-treated males (Fig. S5G). Striking differences in the gross appearance of the GTs between sexes were observed: female GTs exhibited overall normal size and morphology (Fig. 5A, upper panels, compared to controls), whereas littermate male GTs exhibited a spectrum of defects including stunted GT outgrowth, underdeveloped and misshapen glans, and hypospadias (Fig. 5A, lower panels, Fig. S5D). Hematoxylin–eosin (H&E) staining revealed that both male and female GTs showed signs of differentiation defects, but the phenotype is much more severe in male GTs (Fig. 5B to E). Hemorrhaging vessels were apparent in many Cediranib-treated male GTs, and the Cediranib-treated GTs were significantly smaller than controls. In addition, the prospective corpus body (CB) of Cediranib-treated male GT was less well-defined, lacking a distinctive corpus cavernosum structure (Fig. 5E). The urethral folds did not completely fuse at the ventral surface of the GT (Fig. 5E, arrow), resulting in exposure of the urethra and hypospadias. BrdU pulse labeling and subsequent immunohistochemistry (IHC) were performed to quantify cell proliferation in these specimens. Sexual-dimorphic proliferation rates were apparent in control E17.5 GT mesenchyme, in both the CB region (0.001079 ± 4.346e-005 BrdU+ cells/pixel^2^ in male, *N* = 4 vs. 0.0006211 ± 2.044e-005 in female, *N* = 3; *P *= 0.0004) and the prepuce region (0.0009609 ± 5.790e-005 in male, *N* = 4 vs. 0.0005567 ± 1.158e-005 in female, *N* = 3; *P *= 0.0021) (Fig. 5F, G, and J). Daily Cediranib-treatment from E14.5 to E16.5 led to a significant reduction in the number of BrdU+ cells in male GT mesenchyme (Fig. 5I and J) down to levels comparable to females, but minimally affected littermate female embryos (Fig. 5H and J). These data taken together confirmed the in-vitro findings and validated that Cediranib is a potent inhibitor of GT development by inhibiting proliferation and interrupting differentiation in vivo.

### Optimization of siRNA silencing in isolated GTme cells

**Fig. 6.**
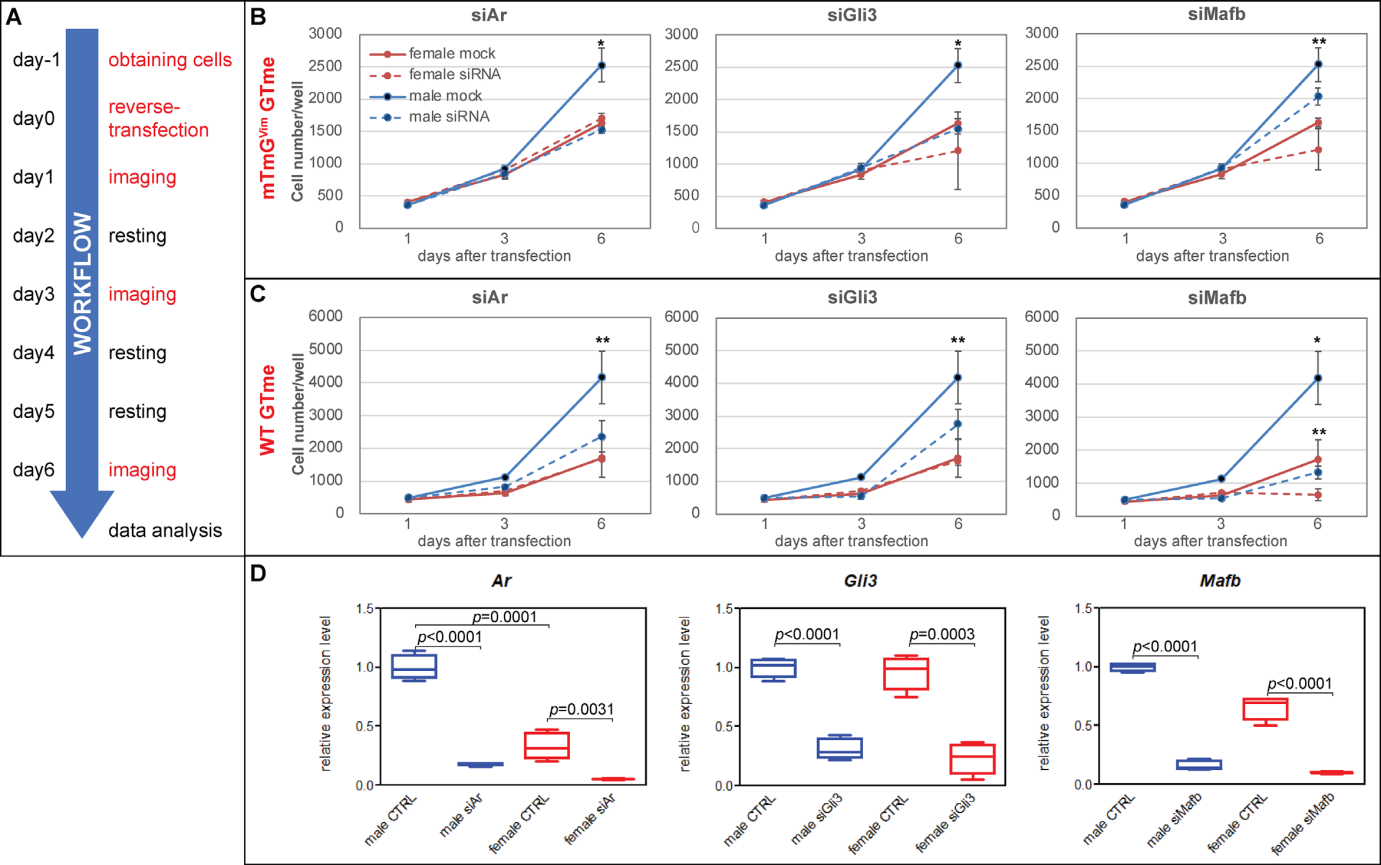
siRNA silencing of genes critical to GTme growth. (A) Experimental design for gene silencing and proliferation assay in isolated GTme cells. (B and C) Growth curves of GTme cells reverse-transfected with siRNA against *Ar, Gli3*, and *Mafb*. *P < 0.01, siRNA vs. mock of same sex; **P < 0.05. (D) Real-time qPCR confirming efficient knockdowns of respective genes. Note the sex-dimorphic expression of *Ar* in the control GTme cells (male 0.9987 ± 0.0526 vs. female 0.3290 ± 0.0568, *N* = 4, *P *= 0.0001).

One goal of establishing the current model is to enable genome-wide large-scale siRNA silencing screening for genes involved in GTme proliferation. We first set out to optimize siRNA transfection protocol by titrating the seeding density of GTme cells and the concentration of DharmaFECT4 transfection reagent in the presence of 20 nM siGLO, a fluorescent nontargeting siRNA to visualize successful transfection (Fig. S6). Greater than 80% transfection efficiency with robust intracellular incorporation of siGlo dye was achieved 48 hours post-transfection when GTme cells were seeded at 500 cells/well (384-well plates) and reverse-transfected with 0.05 µl/well DharmaFECT4 (Fig. S6). To test the feasibility of coupling siRNA silencing and our screening system, we tested a handful of genes that are known to be required in GT outgrowth and differentiation, including *Ar, Gli3*, and *Mafb* (11, 12, 26–28). The experimental design for gene silencing and proliferation assay is illustrated in Fig. 6A. Briefly, E16.5 GTme cells originating from either WT or mTmG*^Vim^* embryos were obtained the day prior to reverse transfection. GTme cells of each sex in three sets of quadruplets were reverse-transfected with validated SilencerSelect siRNA against each target gene, and each set of quadruplet cells were nuclear-stained and imaged on day 1, 3, and 6 post-transfection, respectively. As shown in Fig. 6B and C, silencing of both *Ar* and *Gli3* caused significant reduction in cell number on day 6 specifically in male GTme cells, regardless of the isolation method, but fully down to levels comparable to female when using the mTmG*^Vim^* model. *Mafb* gene silencing impaired GTme cell proliferation in both sexes to various degrees. Satisfactory silencing of respective genes was confirmed by qRT-PCR in mTmG*^Vim^* GTme cells (Fig. 6D). The sex-dimorphic responses of GTme proliferation to the silencing of these positive control genes supports the use of our system for future large-scale siRNA screening.

## Discussion

One of the most prominent differences between male and female external genitalia is their size. Even though the primordial GT in either sex starts off as morphologically similar structures during early developmental stages (E10.5 to E14.5 in mice), it acquires drastically different growth rate and differentiation pathways in a sex-dependent manner afterwards (E15.5—birth) [for review, see ref. (29)]. Sexual differences in GT width and length can be observed as early as E17.5 and E18.5, respectively (29, 30). These events are mainly powered by mesenchymal proliferation and differentiation, where AR and ER are expressed and their signaling cascades are activated. In this study, we developed two isolation methods to obtain enriched and pure GTme cells, and established a high-throughput screening method that recapitulates the sexual-dimorphic growth/differentiation phenotype of GTme cells in vitro.

The crude isolation method involves two steps of enzymatic digestion, and a mechanical separation of epithelial compartments from the tissue rudiments in between. This method yields a cell mixture consisting of ∼70% GTme, and all six subpopulations of GTme were recovered. To improve the purity of the isolated cells, we engineered a *Vim*-rtTA knockin mouse line and generated mTmG^*Vim*^ embryos. One step enzymatic digestion of mTmG^*Vim*^ GT followed by FACS-sorting for GFP+ cells was sufficient to acquire pure GTme cells. GTme cells isolated with either method can proliferate continuously for 10 to 15 days, exhibit similar sex-specific growth rate, and to a large extent, respond similarly to exogenous chemicals. Both methods have their pros and cons. The crude enrichment method uses widely accessible wild-type embryos as the source of isolation; hence, the yields of GTme per litter per sex are in general abundant (roughly 100,000 to 200,000 cells/GT), but this method includes some contaminant epithelial cells that can complicate analysis. The FACS-based isolation method improves the purity dramatically but requires the use of the newly generated *Vim*-rtTA mouse line. Depending on the number of alleles of *Vim*-rtTA and tetO-Cre the stud carries, only a fraction of the embryos will provide GFP+ GTme cells and the rest goes to waste. However, by breeding *Vim*-rtTA and tetO-Cre mice together and performing copy number qPCR on gDNA, it is possible to generate and identify studs homozygous for both transgenes, and breed to females homozygous for mTmG. This would ensure that every embryo in every litter can be used for GTme isolation, and each litter would produce an average of 400,000 to 800,000 pure GTme cells per sex, making downstream large-scale screening feasible. Lastly, comparing the two methods directly can be beneficial. By nature of including some epithelial cells, the crude enrichment method can serendipitously identify modulators that are at least partially driven by epithelial cells when its results are directly compared to the FACS-based pure mesenchymal isolation results. Our findings suggest that this is likely the case when *Mafb* is silenced.

It is vital to note that development of the murine *Vim*-rtTA allele is a critical addition to all subfields of developmental and disease biology, not just genitalia development as demonstrated here. With the appropriate flox allele crossed in alongside tetO-Cre, the *Vim*-rtTA allele herein developed can be used to drive deletion, overexpression, or point mutation of any gene, as well as any flox-based reporter expression, in a time-specific manner using Dox administration at any induction time without affecting endogenous VIM expression. With VIM being arguably the cleanest and most widely expressed mesenchymal marker, the development of a Dox-inducible VIM driver that does not disrupt endogenous expression of VIM represents a powerful tool to distinguish gene function between mesenchymal and epithelial compartments of any developing or diseased organ, with the unique exception of the uterus, which demonstrates MET.

Characterization of the isolated GTme cells by qPCR demonstrated that our isolation method retains all subpopulations of mesenchymal cells, as previously defined by single cell RNA sequencing data (16). Most of these subpopulations appeared to proliferate at similar rates, since marker expression remained largely unchanged throughout the culture period. One exception is the PUO region, where significant elevation in expression of both markers for this subpopulation was observed at the end of culture. Two scenarios may lead to this increase: (1) cells of this population possess a proliferation advantage and over time make up a larger percentage of GTme cells; and (2) these cells continue to proliferate at a similar rate as the rest of the GTme cells, but express much higher levels of the PUO region markers over time. The fact that marker expression for other subpopulations remained at similar levels suggests that scenario 2 is more likely, because otherwise we would expect a concomitant reduction in the markers of other cell types as a result of the increase in the PUO region cells. It is also possible that the dissociation process during GTme isolation disrupted the extracellular matrix, and the lack of signaling and support from endodermal and ectodermal cells in GTme culture caused the cells to behave differentially than they would in vivo. The sex-dimorphic proliferation capacity exhibited by the isolated GTme cells prompted us to hypothesize that intrinsic AR signaling was the driving force. Indeed, supplementing the cells with MT significantly elevated the proliferation rate of female GTme cells, whereas AR inhibitor FLUT antagonized this effect. It is noteworthy that we also tested testosterone and dihydrotestosterone at 10 nM, which did not appear to enhance female GTme proliferation (Fig. S7). The differential effects of various testosterone metabolites on GT development have been shown both in-vivo and in-organ culture system previously (31), but the underlying mechanisms remain unclear. Nevertheless, the presence of sex-dimorphic proliferation in isolated GTme cells within the assayed time demonstrates the power of this model.

High-throughput screening is a versatile method that allows automated testing of large numbers of biological, genetic, chemical, and/or pharmacological agents. We previously developed a high-throughput screening tool to quantitatively monitor the human decidualization process in vitro with the use of an engineered fluorescent human endometrial stromal cell line (20). In the current study, we adapted the previous readout system and used an image-based method to monitor GTme proliferation and differentiation in vitro. Traditional proliferation assays used for cultured cells include DNA synthesis assays, metabolic assays, luminescent assays, and cell counting assays. Metabolic assays (MTT, WST1, XTT, etc.) and luminescent assays (bioluminescent ATP luciferase, etc.) may not accurately reflect proliferation rates due to a miscorrelation of metabolic activity and cell number, and they do not provide accurate readings when cell density is low. DNA synthesis assay involves treating cells with fluorescent BrdU/EdU and subsequent microscopic imaging, which adds time and cost to the screening process. Traditional cell counting assays require trypsinization of cultured cells, counter-staining for live cells (Trypan Blue, etc.), followed by manual counting on a hematocytometer or automated cell counter, and are therefore not suitable for a high-throughput setup. The cell counting method we adopted involves one simple DNA counter-stain step 15 minutes prior to imaging, and the automated IN Cell Analyzer allows rapid imaging of the entire 384-well plate in minutes. Subsequent automated image analysis not only allows precise reading of cell count for every well, it also measures and reports nuclear size. This parameter provides additional information on the health and developmental stage of the GTme cells, which can be used to determine the different types of cellular responses a biological/chemical molecule elicits.

With this powerful screening tool in hand, we tested a 438-CPD kinase inhibitor library and uncovered 82 chemicals that either promote or impair GTme cell proliferation in vitro, in either one or both sexes. Fisher’s exact test identified mTOR and PLK inhibitors as the most enriched families of inhibitors identified in our screen, and EGFR inhibitors as the most disenriched. The mTOR, belongs to the phosphatidylinositol 3-kinase–related family of kinases, and forms two distinct complexes named mTOR complex 1 (mTORC1) and 2 (mTORC2) with several other protein partners [for review, see ref. (32)]. mTORC1 signaling pathway promotes protein synthesis by directly phosphorylating S6 kinase 1 and 4E-BP1, which in turn increases transcription and initiates translation [for review, see (33)]. mTORC1 also directly controls lipid biosynthesis, a key step to generate cytoplasmic membrane to ensure cell proliferation (34). mTORC2, on the other hand, phosphorylates substrates like AKT and SGK, which subsequently regulate cellular processes such as apoptosis, ion transport, metabolism, and cell growth (35, 36). Transcripts of *Mtor* are detected in the E14.5 GT, at higher levels in the ectoderm and to a less extent in the dorsal mesenchyme [GenePaint.org (37)]. A recent study using a chemical-induced rat hypospadias model suggested that decreased mTOR activity is associated with hypospadias, possibly through increased autophagy (38). Functions of mTOR pathway in the GT remains unclear due to early embryonic lethality of the *Mtor* knockout model (39), and our findings demand further investigation of its role during GT development. PLK is a serine/threonine protein kinase that plays essential roles in mitosis, both at the onset of G2/M transition and during cytokinesis [for review, see ref. (40)]. PLK1, the most thoroughly studied PLK, is highly expressed in rapidly dividing cells of adult tissues and during embryogenesis. Basal activity of PLK1 has also been shown to regulate DNA replication, under cellular stress (41). Transcripts of *Plk1* and *Plk4* appear to be abundant in embryonic GT mesenchyme [GenePaint.org (37)], and their involvements in GT development require further investigation.

To validate our in-vitro findings, we tested one of the hits that elicited sexual-dimorphic responses in GTme proliferation in mice. Cediranib (AZD2171, AstraZeneca) is a potent inhibitor of VEGFR tyrosine kinase that has demonstrated efficacy as an antineoplastic drug in various cancers (42). It is currently undergoing or has previously completed preclinical studies for treatment of ovarian, endometrial and other types of solid tumors (43–45) (NCT04487587, NCT00942877). Due to the criteria for patient eligibility, its effects on pregnant patients and their fetuses remain unclear. In-vitro cell proliferation assay demonstrated that Cediranib impaired male GTme proliferation while minimally affecting female counterpart GTme. Three doses of daily Cediranib administration resulted in a plethora of GT malformations ranging from hypospadias and abnormal glans, to stunted GT outgrowth in male embryos. Quantification of cellular proliferation by BrdU incorporation confirmed significant reductions in a male-specific manner. These data clearly demonstrate the feasibility of translating in-vitro data acquired using our screening system to animal studies.

Lastly, we optimized gene silencing in isolated GTme cells and coupled it with the proliferation assay. Silencing of genes (*Ar* and *Gli3*) known to play essential roles in sex differentiation of external genitalia (11, 28, 46), resulted in male-specific inhibition of GTme proliferation. Knocking-down *Mafb*, a pivotal mediator of AR, appeared to impair cell proliferation in both sexes, even though genetic ablation of the gene does not cause any apparent phenotype in females (27). Intriguingly, GTme obtained by the crude isolation appeared to have a more robust response to *Mafb*-silencing–induced cell growth inhibition, suggesting cell-type–specific responses to external stimuli and/or interactions between different cell types playing a role in cell proliferation.

Despite being among the most prevalent types of congenital structural anomalies, the etiologies of most cases of genitalia anomalies go unsolved, and treatment options are almost entirely limited to complex surgeries. Only a handful of research labs across the Unites States specialize in identifying genetic and environmental modulators of the genitalia during its development and differentiation, and because of this, only a small number of new chemical and genetic regulators are identified each year. The crude enrichment screening system described herein is designed with accessibility in mind such that any lab can utilize it. Even without the high-throughput imaging system used in this study, viability stains with cell count can be used for small-scale screens. The pure isolation protocol we developed is designed with speed in mind. As demonstrated in our proof-of-concept experiments, it can rapidly screen hundreds if not thousands of potential modulators of genitalia mesenchymal growth and differentiation. Together, these tools offer the research community efficient ways to rapidly identify potential etiologies of genital anomalies, both environmental and genetic, with the goal of diversifying treatment options for affected patients.

## Materials and Methods

### Generation of *Vim*-rtTA knock-in mice

A donor plasmid was constructed by inserting a SP163 enhancer, the rtTA coding sequence, and a 2A linker into 1kb-long 5’- and 3’-homologous arms targeting the mouse *Vim* genomic locus. Single-guide RNA (sgRNA) was designed and validated by the Genome Engineering an iPSC Center at Washington University. Effectiveness of donor-mediated genomic editing was validated in mouse N2a cell line in the presence of sgRNA and Cas9 nuclease. The donor plasmid, sgRNA and Cas9 mRNA were simultaneously micro-injected into single cell–stage mouse embryos of hybrid genetic background, and transferred back into pseudopregnant female recipients at the Micro-Injection Core (Department of Pathology & Immunology, Washington University School of Medicine). Junctional PCR on both 5’- and 3’-ends was performed to confirm insertion of the construct using primers listed in Table S3 with extracted genomic DNAs.

### Mouse maintenance and drug treatment

All mice used in this study were maintained in a barrier facility at Washington University School of Medicine, MO, following the institution’s regulations with an approved protocol (#21–0438). Wild-type Swiss outbred mice were purchased from JAX (#034,608). Confirmed *Vim*-rtTA founder was bred to tetO-Cre transgenic mouse (21), and offspring *Vim*-rtTA; tetO-Cre studs were bred to ROSA26^mTmG^ females [(22), JAX #007,676] to generate embryos for the study. Pregnant ROSA26^mTmG^ females received 20 mg/ml doxycycline (Sigma #D9891) dissolved in water at a dosage of 0.05 g/kg via oral gavaging at E10.5 to induce Cre expression. A 25 mg/ml Cediranib (Selleck Chemicals #S1017) DMSO stock was diluted in corn oil to make a 1.25 mg/ml working solution, which was given daily to pregnant wild-type females from E14.5 to E16.5 at a dosage of 5 mg/kg via oral gavaging.

### GTme isolation and culture

For wild-type GTme isolation, E16.5 embryos were collected in cold sterile Hank’s Balanced Salt Solution (HBSS, ThermoFisher #14,175). Sex of embryos was determined by gonadotyping, and embryonic GT was carefully dissected with a pair of Vannas Scissors and pooled by sex. Each GT was cut in half along the urethra with a scalpel. Once male and female GT tissues were individually processed and collected in 1 ml HBSS, trypsin (Sigma #T1426) was added to a final concentration of 0.25%. Tissues were incubated at room temperature on an orbital shaker for 30 minutes. The content was subsequently poured into a sterile P6 dish containing fresh cold HBSS. Working with one piece at a time, epithelial tissues were extracted carefully with fine forceps, and remaining mesenchymal tissues were collected in a tube containing fresh HBSS by pipetting. Trypsin and collagenase (Sigma #C0130) were added to final concentrations of 0.25% and 1 mg/ml, respectively, and incubated again for 30 minutes. The tissue remnants were briefly spun down and resuspended in GTme culture medium (phenol red-free DMEM/F12, 7.5% charcoal-stripped FBS, 1× nonessential amino acids, 1× antibiotic–antimycotic). Mesenchymal cells were dissociated into single cell suspension by repetitive pipetting followed by filtering through a 70 µm nylon filter, and seeded onto polylysine-coated flasks. For mTmG^*Vim*^ GTme isolation, pooled GT of same sex were bisected, and digested in 0.25% trypsin and 1 mg/ml collagenase at room temperature for 60 minutes. Single cell suspension of entire GT was then obtained by repetitive pipetting followed by filtering, and plated onto polylysine-coated flasks. The following day, attached cells were trypsinized and resuspended in culture medium, and subject to FACS sorting on a MoFlo cytometer at the Siteman Flow Cytometry Core at Washington University School of Medicine. GFP+ GTme cells were collected, counted, and seeded for subsequent experiments. All cell culture was carried out in humified incubator at 37°C with 5% CO_2_. MT (Sigma M7252) and FLUT (Sigma F9397) were dissolved in 100% ethanol to make a 1000× stock solution, and added to culture medium at indicated final concentrations.

### Image-based screening assay

GTme cells were seeded in 384-well plates at 300 to 500 cells/well/50 μl in quadruplets and imaged at indicated time. Before imaging, cells were treated with Hoechst 33,342 (10 μg/ml, Sigma B2261) for 15 minutes at 37°C. Plates were imaged immediately following staining in IN Cell Analyzer 2,000 automated microscope (GE Healthcare) with 10× objective at four fields per well using DAPI filter. Batch analyses by IN Cell Developer Toolbox software were employed to automatically capture and analyze nuclear size of each cell, and output the mean nuclear size and total cell number of each well in Excel format. For kinase inhibitor screening, cells were treated with 250 nM CPD (Selleck Chemicals L1200) for 6 days. Each plate assaying for approximately 80 CPDs was analyzed independently. For each well, an absolute deviation was calculated as |[cell count]_well_– [cell count]_Median of Plate_|, and an MAD was obtained for each plate. Those with [cell count]_CPD_ above ([cell count]_Median of Plate_ + 3MAD) were identified as hyperhits, and those below ([cell count]_Median of Plate_–3MAD) hypohits. For RNAi silencing, trypsinized GTme cells were seeded on 384-well plates at 500 cells/50 μl medium/well. Aliquots of 5 μl phenol-red free Opti-MEM (Thermo Fisher, #11,058,021) containing 0.05 μl DharmaFECT4 transfection reagent (Dharmacon, #T-2004-04) and 0.05 μl 20 μM siRNA stock (Table S3) were added to each well, to a final concentration of 20 nM siRNA. Each condition was performed in three sets of technical quadruplets, and each set assayed at 1, 3, and 6 days after reverse transfection, respectively.

### RNA isolation and real-time RT-PCR

RNA was extracted with Qiagen RNAeasy mini kit following the manufacturer’s instructions (Qiagen). Reverse transcription was performed using the high-capacity cDNA reverse transcription kit (Applied Biosystems Inc., ABI), and qPCR performed on QuantStudio 6 real-time PCR System (ABI) using PowerUp™ SYBR™ Green Master Mix (ABI). All results were repeated in three biological replicates unless otherwise specified and relative gene expression changes were determined by delta–delta *Ct* method (normalized to housekeeping gene, *Rpl7*).

### BrdU treatment, histology, IHC, and IF

Mice received i.p. injection of 10 mg/ml BrdU (Sigma B5002) dissolved in sterile saline at 5 μl/g b.w. 3 hours prior to euthanization. Tissues fixed in Bouin’s fixative overnight at 4°C were processed for serial dehydration and embedding in paraffin. Eight micron sections were used for H&E staining, IHC and IF as described previously (47). Antibodies were diluted in blocking solution (1% BSA, 3% normal goat serum in PBS) and a list of dilutions and commercial sources can be found in Table S3.

### Testosterone measurements

A transverse cut was made across the thorax of male embryos and blood was collected by 200 μl capillary pipets (∼50 to 80 μl per animal). Blood samples were allowed to clot for 15 minutes at room temperature, followed by centrifugation at 3,000 rpm at 4°C for 10 minutes to separate serum from plasma, and stored at −80°C. Whole testes were snap frozen and stored at −80°C until ready for assay. Free testosterone levels of plasma and testes lysates were performed using the testosterone ELISA kit (Cayman Chemical, #582,701) following the manufacturer’s instructions. Four male embryos of each treatment group were measured and analyzed.

### Flowcytometry

Isolated WT GTme cells were analyzed for the presence of mesenchymal and epithelial markers using FACS. After brief fixation in 4% PFA, individual cell aliquots (2 × 10^5^) were washed in FACS buffer (1×PBS, 0.5% BSA, 0.1% NaN3) and incubated with isotype/antibody cocktails overnight at 4°C (antibody information listed in Table S3). The cells were then washed with FACS buffer and collected by centrifugation. Stained cell pellets were resuspended in 0.5 ml FACS buffer and analyzed on FASCan (BD BioSciences). Isolated mTmG^*Vim*^ GT cells were allowed to attach to polylysine-coated flasks overnight. The following day, cells were trypsinized, centrifuged then resuspended in culture medium, and suspended cells were sorted on MoFlo Cytometer gated for GFP at the Siteman Flow Cytometry Core Lab at the Washington University School of Medicine.

### Statistical analysis

All experimental groups contained three biological and four technical replicates, if not specified otherwise. Two-tailed Student’s *t*-test assuming unequal variance was performed to compare means of the experimental groups. Data are presented as mean ± SD, and *P*-value less than 0.05 was considered statistically significant.

## Supplementary Material

pgac300_Supplemental_File

## Data Availability

All study data are included in the article and/or supplementary material.
