# Supplementary material for: Development of an in-vitro high-throughput screening system to identify modulators of genitalia development
Source: PNAS Nexus. 2022 Dec 28;2(1):pgac300. doi: 10.1093/pnasnexus/pgac300 (PMC9832959; doi:10.1093/pnasnexus/pgac300)
Supplement: pgac300_Supplemental_File [file pgac300_supplemental_file.pdf]

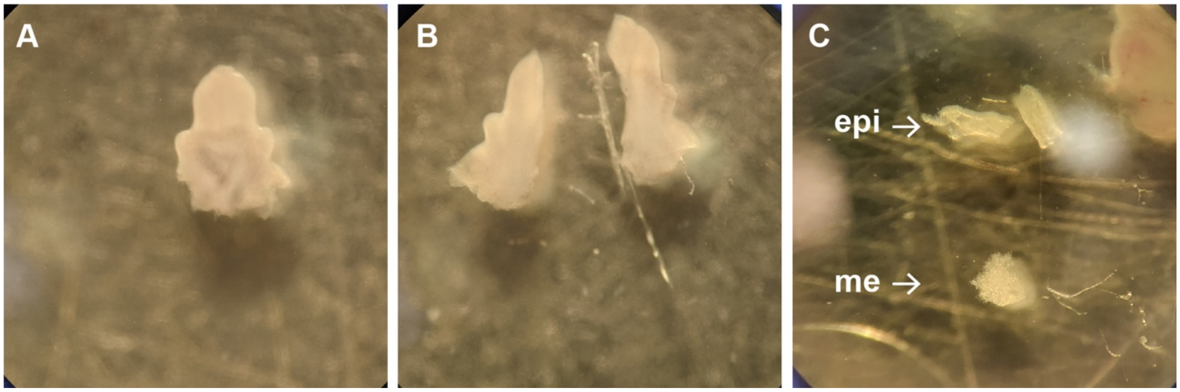

Figure S1. Gross morphology of E16.5 GT right after dissection (A), after being bisected (B), and after the initial trypsin digestion (C). Chunks of mesenchymal tissues (me) were further digested in collagenase/trypsin after being separated from epithelial sheets (epi).

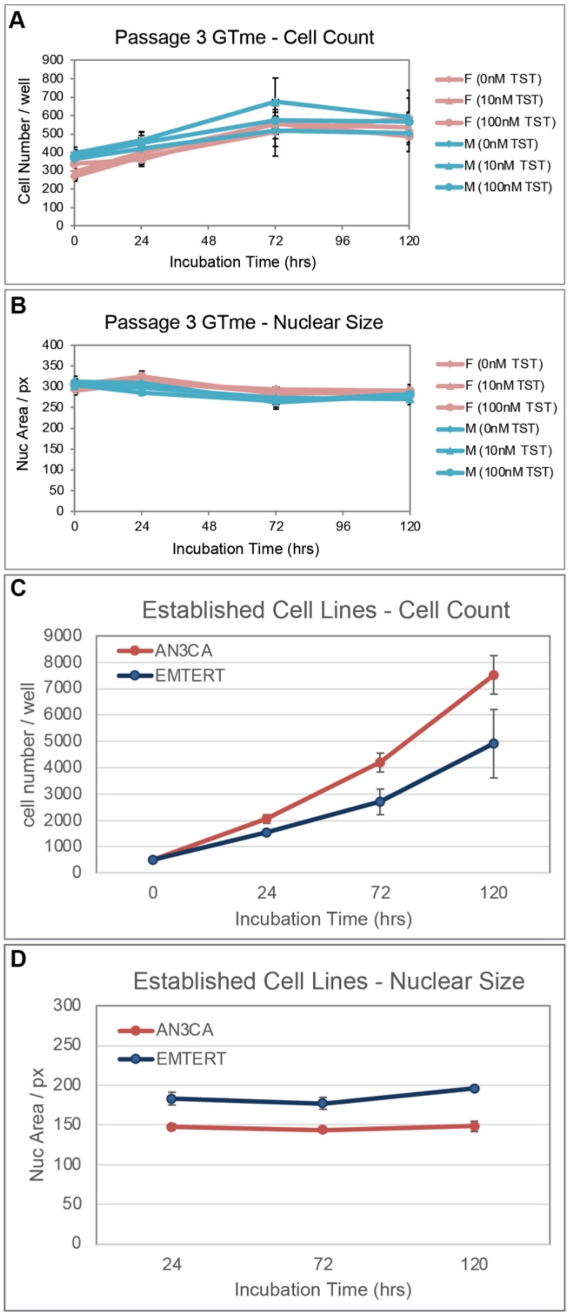

Figure S2. GTme cells lose growth capacity over time. Growth curves (A) and measured nuclear size over time (B) of passage three GTme cells with or without testosterone supplement compared with those of established cell lines AN3CA and EMTERT (C, D).

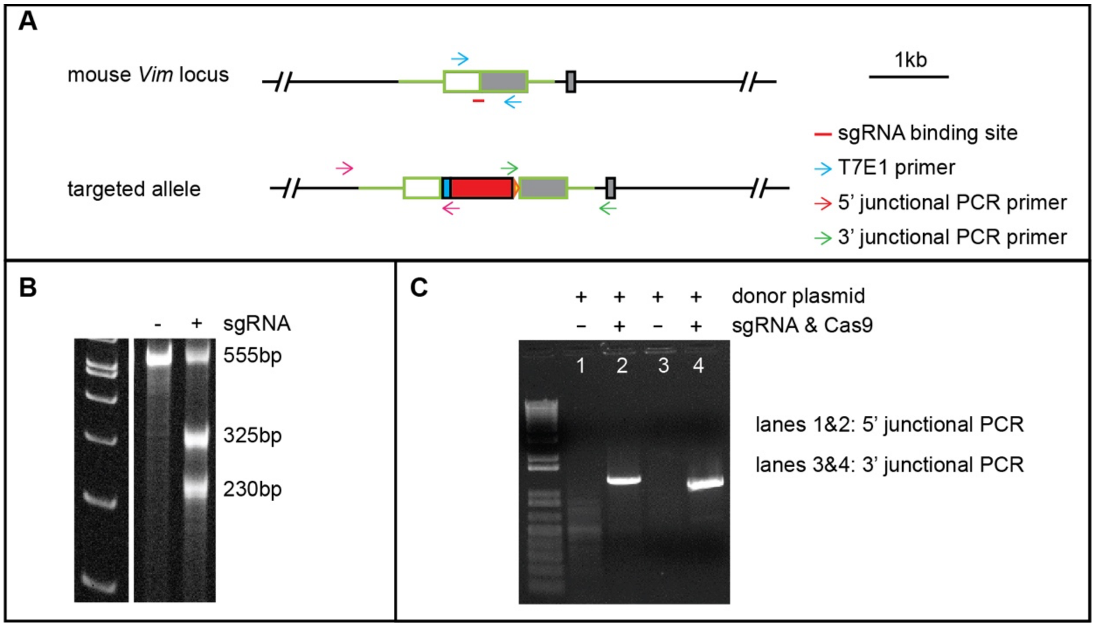

Figure S3. Validation of sgRNA and donor plasmid in vitro. (A) Schematic illustration of primer design for T7E1 assay and junctional PCR. (B) Successful NHEJ events were detected in N2A cells transfected with designed sgRNA. (C) Successful homologous recombination was achieved in N2A cells co-transfected with donor plasmid, sgRNA and Cas9.

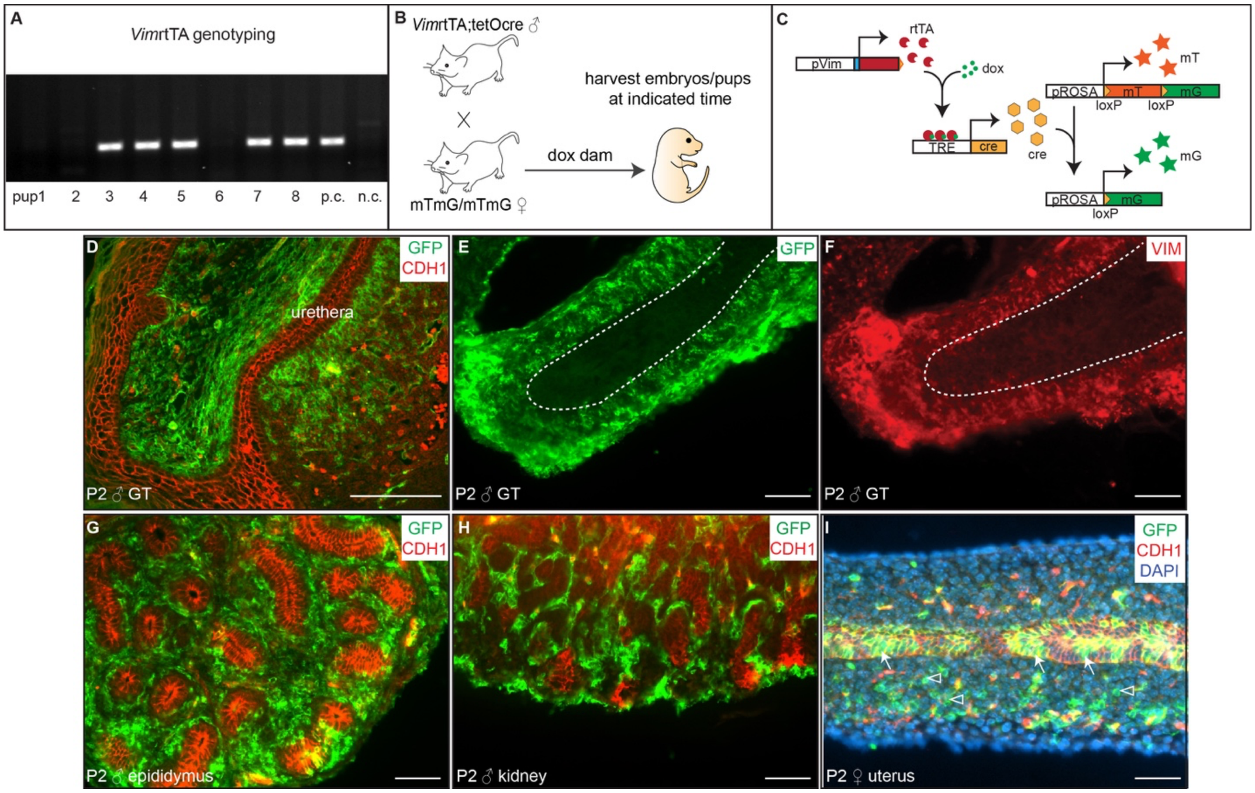

Figure S4 Generation and characterization of *Vim*-rtTA mouse. (A) PCR genotyping of littermates sired by the *Vim*-rtTA founder using primers illustrated in Fig. 3B. p.c. positive control; n.c. negative control. (B) Mating scheme to obtain embryos harboring *Vim*-rtTA, tetO-Cre and mTmG alleles. (C) A schematic of Vim-rtTA;tetO-Cre;mTmG system. (D-I) Immunofluorescence of GFP, CDH1 and/or VIM in various organs of postnatal day 2 mTmG^{Vim} mice. Homogeneous GFP staining (green) was detected in the GT (D,E), epididymis (G) and kidney (H) mesenchyme, and was excluded from CDH1-expresing epithelia (red). In the GT, GFP expression recapitulates that of the endogenous VIM exclusively in the mesenchyme. Dotted lines outlined the urethra (E, F). (I) In the neonatal uterus, sporadic GFP+ cells were detected in the stroma (open arrow heads), and GFP+ cells (arrows, green) and transitional cells (yellow) were also detected in the luminal epithelium. Scale bars: 100 µm.

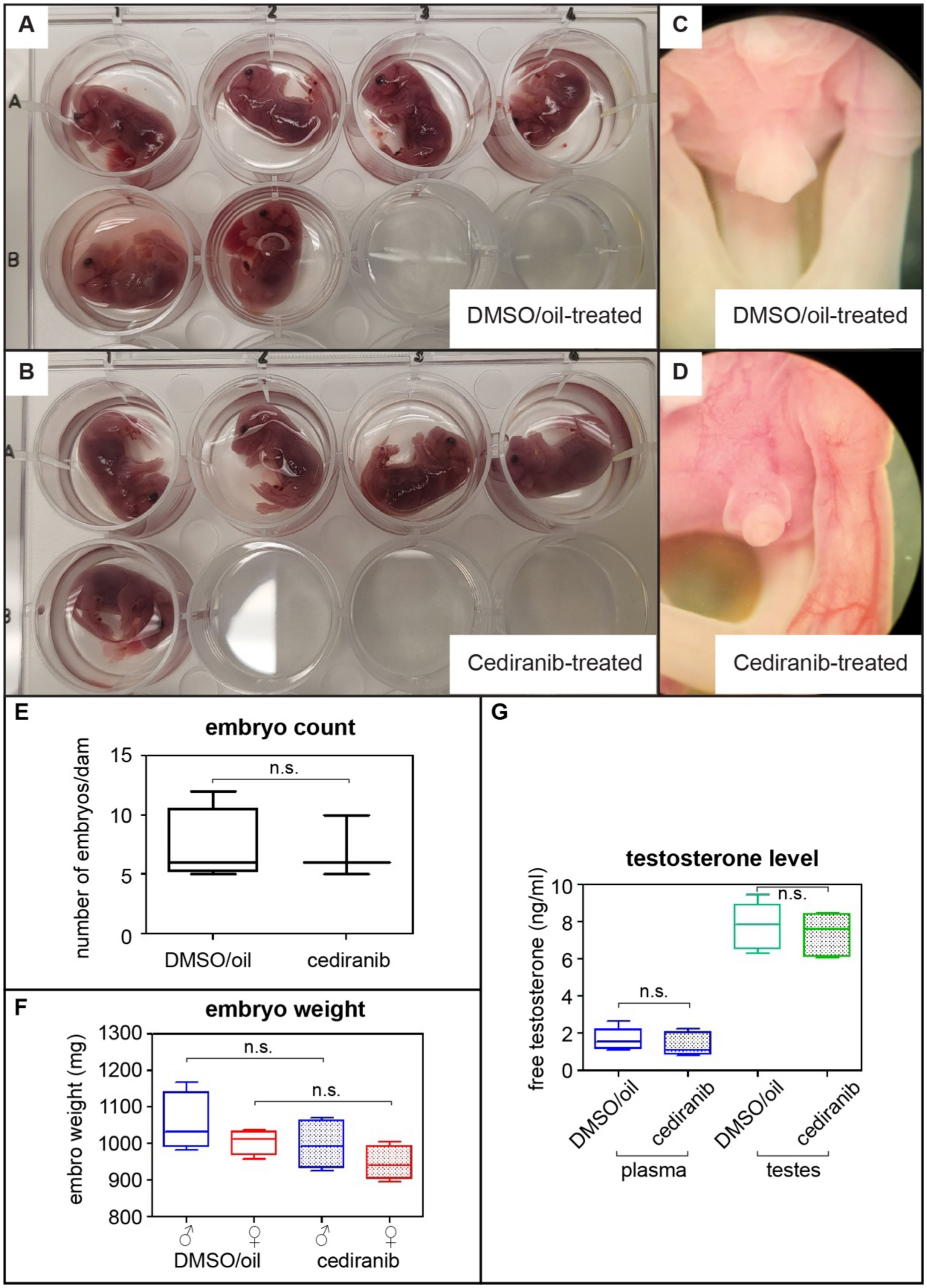

Figure S5. Evaluation of cediranib-treated embryos. (A, B) Litters of E17.5 embryos from dams that received daily oral gavaging of either DMSO/oil (A) or cediranib (B) from E14.5-E16.5 showed comparable embryo number and size. (C, D) Closer view of a DMSO/oil-treated male embryo (C) and a cediranib-treated counterpart (D). Note the normal size of other tissues (legs

and tail) but marked reduced GT size in cediranib-treated embryo. (E) DMSO/oil-treated dams produced 7.25 ± 1.60 pups, n=4; while cediranib-treated dams produced 7.00 ± 1.53 pups, n=3; p=0.917. (F) Cediranib treatment did not affect embryo weight. DMSO/oil male, 1054 ± 41.02 mg, n=14, vs. cediranib male, 996.1 ± 34.82 mg, n=12, p=0.3242; DMSO/oil female, 1004 ± 17.17 mg, n=15, vs. cediranib female, 945.6 ± 23.45 mg, n=9, p=0.0895. (G) Free testosterone level measured by ELISA revealed no significant difference in either embryonic plasma or whole testis lysates of treated male embryos. N=4 for each of the following four groups: DMSO/oil plasma, 1683 ± 239.3 pg/ml vs. cediranib plasma, 1359 ± 249.8 pg/ml, p=0.3710; DMSO/oil testes, 7812 ± 497.8 pg/ml vs. cediranib testes, 7380 ± 433.7 pg/ml, p=0.0895.

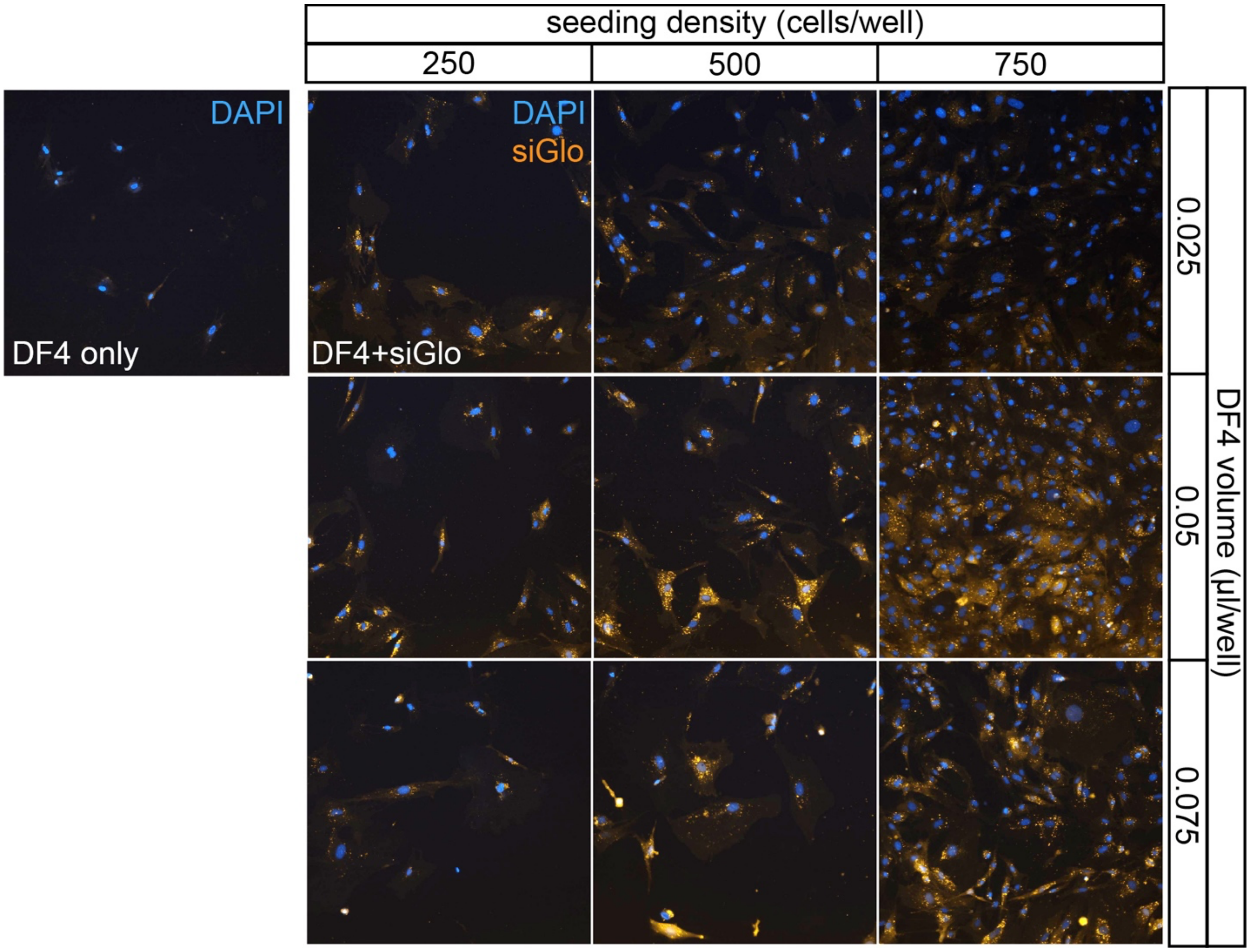

Figure S6. siRNA transfection optimization in GTme cells. GTme cells were seeded at indicated density and reverse-transfected with indicated amount of DharmaFECT4 reagent and siGlo at a final concentration of 20nM. Fluorescence microscopy was utilized to visualize incorporation of siGlo 48 hours after transfection. High transfection efficiency and minimal cytotoxicity were achieved with 0.05 µl transfection reagent/well, and seeding density of 500/well appeared to have the highest per cell intracellular dye incorporation.

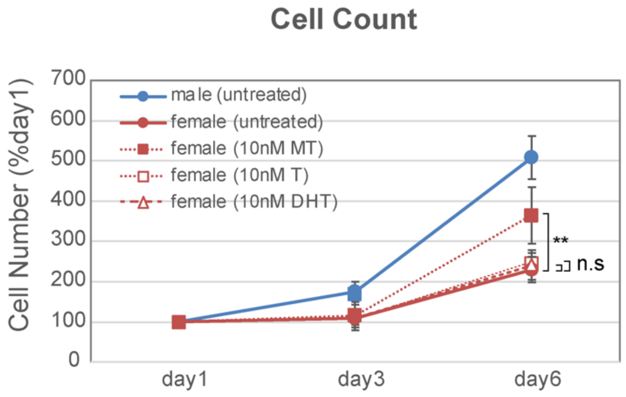

Figure S7. Treatments of testosterone (T) and dihydrotestosterone (DHT) at 10 nM did not boost female GTme proliferation as did methyltestosterone (MT). ** p<0.01; n.s, not significant.

Table S2. Top 5 signaling pathways revealed by PANTHER analysis

PANTHER pathways	Fold Enrichment	raw P value	FDR
**Angiogenesis**	51.74	1.63E-12	2.62E-10
**CCKR signaling map**	49.72	6.74E-11	5.42E-09
**VEGF signaling pathway**	86.4	3.64E-09	1.96E-07
**Integrin signaling pathway**	36.56	1.14E-08	4.57E-07
**Gonadotrophin-releasing hormone receptor pathway**	29.56	3.89E-08	1.04E-06

Table S3. Oligos and antibody information

**Oligo Information**				
**Sequence Name**	**Sequence (5'->3')**			
Dkk2 F	CTGATGCGGGTCAAGGATTC			
Dkk2 R	CTCCCCTCCTAGAGAGGACT			
Gata6 F	TTGCTCCGGTAACAGCAGTG			
Gata6 R	GTGGTCGCTTGTGTAGAAGGA			
Klf6 F	TTTCTGCTCGGACTCCTGAT			
Klf6 R	TTCCTGGAAGATGCTACACATTG			
Runx1 F	TTTCAAGGTACTCCTGCCTG			
Runx1 R	CAGTGAGAAGGACCAGAGAC			
Klf4 F	GTGCCCCGACTAACCGTTG			
Klf4 R	GTCGTTGAACTCCTCGGTCT			
Zfhx4 F	GACTCTCCTCCTATCTCAAG			
Zfhx4 R	CGTTTTCAGGTTGTCATGTG			
Foxf1 F	AATGCACACATCTCCCTTCC			
Foxf1 R	TTCACCTGTGATTGCTGGAG			
Tcf21 F	CTCCCTGAAAGTGGACTCCAA			
Tcf21 R	CGGGCTTTTCTTAGTGGGC			
Cdk6 F	GGCGTACCCACAGAAACCATA			
Cdk6 R	AGGTAAGGGCCATCTGAAAACT			
Trps1 F	TTGCATTCTGTAGTGGCGTT			
Trps1 R	GAGACTCCCGCATCTGAGAA			
Col8a1 F	ACTCTGTCAGACTCATTCAGG			
Col8a1 R	CAAAGGCATGTGAGGGACTTG			
Pid1 F	AGGCAAGGACTCATAGTGGC			
Pid1 F	CCAGTGTGTGTTTCTTCCAG			
Tbx18 F	GTACCTGGCTTGGCACGAC			
Tbx18 F	GCATTGCTGGAAACATGCG			
Vim 5'junctional F	CGAGATCACTGGGCTTGCTT			
Vim 5'junctional R	GGCTCCTCTCCCTTCTGGA			
Vim 3'junctional F	TAGACATGCTCCCAGCCGAT			
Vim 3'junctional F	CACACCCAACCACAAATGCC			
Vim-rtTA F	TCCCTTGTTGCAGTTTTTCC			
Vim-rtTA R	CTCCCAGCTTTTGAGCGAGTTTCCTTGTCG			
**Antibody Information**				
**Name**	**Vendor**	**Cat#**	**dilution**	
E-Cadherin (24E10) Rabbit mAb (Ale	Cell Signaling	3199	1:100	
Vimentin (D21H3) XP® Rabbit mAb (	Cell Signaling	9856	1:100	
Rabbit (DA1E) mAb IgG XP® Isotype	Cell Signaling	2975	1:200	
Rabbit (DA1E) mAb IgG XP® Isotype	Cell Signaling	2985	1:200	
Vimentin (D21H3) XP® Rabbit mAb	Cell Signaling	5741	1:300	
Mouse Anti-E-Cadherin (CDH1)	Bdbiosciences	610181	1:300	
GFP Polyclonal Antibody	ThermoFisher	A11122	1:200	
Goat anti-Mouse IgG Secondary Ant	ThermoFisher	A32723	1:500	
Goat anti-Rabbit IgG Secondary Ant	ThermoFisher	A32740	1:500	
siRNA				
gene	vendor	catelog number	Identifier	Sequence (if available)
Ar	Thermo Fisher	4390771	n393529	
Gli3	Dharmacon	J-045798-05		GAACAACCCUAGUCAAGGA
Mafb	Dharmacon	J-059035-09		CCUACAAGGUCAAGUGCGA
